# The impact of sea buckthorn oil fatty acids on human health

**DOI:** 10.1186/s12944-019-1065-9

**Published:** 2019-06-22

**Authors:** Marta Solà Marsiñach, Aleix Pellejero Cuenca

**Affiliations:** 1 Departament de Bioquímica i Biotecnologia, Universitat Rovira i Virgili, Crta. de Valls, s/n, 43007 Tarragona, Spain; 2 Medicine Faculty - Universitat Autònoma de Barcelona, Passeig de la Vall d’Hebron, 119-129, 08035 Barcelona, Spain

**Keywords:** Fatty acids, Sea buckthorn, Omega-3, Omega-6, Clinical applications, Human health, Plant, Omega-7, *Hippophae rhamnoides*, Omega-9

## Abstract

The beneficial properties of fatty acids have been undervalued for several years. In contraposition, new studies reveal that fatty acids have an essential role for human health. The aim of the study is to demonstrate the clinical applications of fatty acids present in sea buckthorn oil. The composition of fatty acids found in sea buckthorn (*Hippophae rhamnoides)* oil is unique for this species, presenting a vast range of health benefits for humans and therefore it is highly valued by both biomedicine and the cosmetic industry. In this way, we will see the clinical effect of monounsaturated, polyunsaturated and saturated fatty acids that constitute sea buckthorn oil and how they contribute to the correct function of the organism. Despite there being studies that support the positive effects of sea buckthorn fatty acids, they are limited. Hence, most of the results obtained in this review are from studies of isolated fatty acids instead of fatty acids extracted from sea buckthorn oil. These facts permit to demonstrate the effect of sea buckthorn fatty acids separately but we lost the possibility of detecting a synergic effect of all of them. More studies are necessary to certify the clinical application of the fatty acids present in sea buckthorn oil as well as discovering possible synergies between them.

## Introduction

For several decades, the beneficial properties of fatty acids have been largely ignored. In contrast, this paper will review the contributions made by several researchers who have shed light over the fact that the fatty acid group is actually large and diverse, as well as greatly beneficial for human health.

From a nutritional standpoint, fatty acids have an important role in several metabolic and structural functions. They are indispensable compounds of the cell membranes, are responsible for the transport of vitamins, and regulate the concentration of lipids in plasma. Furthermore, fatty acids produce a number of precursors such as eicosanoides, decosanoides, steroid hormones and biliary acid, all of them fundamental for the adequate functioning of the metabolism. In addition, they are the most important energetic nutrient –it is currently recommended that at least 20% of the total energy intake should derive from lipids–.

As already mentioned, a number of studies have revealed that fatty acids are very diverse, each type of them having different effects over the organism. So far, the fatty acids found to be most beneficial are those presenting an even number of carbons (4 to 30) and instaurations, which are mainly found in marine species. However, lately it has also been highlighted that some plants, such as sea buckthorn, also contain abundant and valuable fatty acids (especially in their seeds) with high nutritional value, as well as useful for several clinical applications. In particular, sea buckthorn is a perfect natural vegetable source to obtain a wide range of fatty acids, especially essential and unsaturated fatty acids, both the most beneficial for human health. It is a plant that is composed of different bioactive compounds, which may also be found in other medicinal plants. The characteristic that makes sea buckthorn unique is the qualitative and quantitative composition of its fatty acids, particularly the presence of the fatty acid omega-7 group, higher than in any other plant.

Sea buckthorn (*Hippophae*) is a hardy bush of the *Elaegnaceae* family classified into nine subspecies, from which *Hipopphae rhamnoides L. subsp sinensis* and *Hippophae rhamnoides L. subsp rhamnoides* are the most applied to commercial purposes [1]. It is a deciduous spiny species distributed all over the temperate zone of Asia and Europe, as well as over subtropical zones at high altitude [2]. Its products, particularly the oil obtained from the seed and the soft parts of the plant, contain an interesting composition of lipophilic compounds. In relation to the oil composition, sea buckthorn is characterized by a unique mixture of bioactive components, being one of them the fatty acids. In general, the oil obtained from seed is rich in omega-3 and omega-6 fatty acids, while in the pulp oils are predominantly fatty acids from the omega-7 group [3] (Table 1). Although the prevalence of fatty acids in the different parts is well established, there could be variations depending on the subspecies, harvesting time and method of isolation [4]. Several mechanisms of oil extraction are used, solvent extraction using hexane and supercritical CO_2_ being the most common in industrial and laboratory scale [5]. In any case, several studies of fatty acids contained in sea buckthorn oil reveal that it may play an important role in very different aspects in relation to the human body, such as cardiovascular disorders, as a stimulator of the immune system, as well as promoting cognitive functions and bone health [6]. Moreover, it may be important to improve various skin conditions such as atopic dermatitis [2], acne skin [7, 8] or psoriasis [9].

**Table 1:** Fatty acid composition of oils from seeds, whole berries and pulp/peel of Sea buckthorn of different origins [4]

Fatty acid	Seed (%)	Berry (%)	Pulp/peel (%)
	*subsp. sinensis*		
Palmitoleic acid	0	33.6	27.2
Linoleic acid	41	18.6	12
Linolenic acid	26.6	11.2	7.1
Oleic acid	19.4	17.6	17.1
Palmitic acid	8.8	22.9	26.7
Stearic acid	2.5	1.5	1.3
Vaccenic acid	2.2	6.7	8.1
	*subsp. rhamnoides*		
Palmitoleic acid	0	26	32.8
Linoleic acid	39	15.3	9
Linolenic acid	30.6	8.8	3.2
Oleic acid	17.2	17.2	17.3
Palmitic acid	7.4	23.7	27.4
Stearic acid	3	1.2	0.7
Vaccenic acid	2.8	7.7	9.1

Means obtained from Yang (2002) [4]

### Objectives

The aim of this review is to demonstrate the clinical application of fatty acids present in sea buckthorn oil. Sea buckthorn was the source selected to demonstrate these positive effects due to its variety and unusual fatty acids in its composition compared to other plants. In general, the studies focused on the fatty acid from sea buckthorn oil are limited and, for this reason, in most cases it has been necessary to classify the different fatty acids presents in the oil composition and reveal their benefits separately Hence, the review will show the clinical application of fatty acids that sea buckthorn oil composes independently of its origin.

## Methods

Systematic searches were conducted using the PubMed database without any restriction in year of publication. Keywords used include “sea buckthorn”, “sea buckthorn oil”, “palmitoleic acid”, “linoleic acid”, “linolenic acid”, “alpha-linolenic acid”, “oleic acid” and “gamma-linolenic acid”. The criteria for inclusion were (1) *in vivo* and in vitro clinical trials and reviews published in English and (2) research studies focused in any health benefits of the fatty acids of sea buckthorn oil.

## Results

**Fig. 1:**
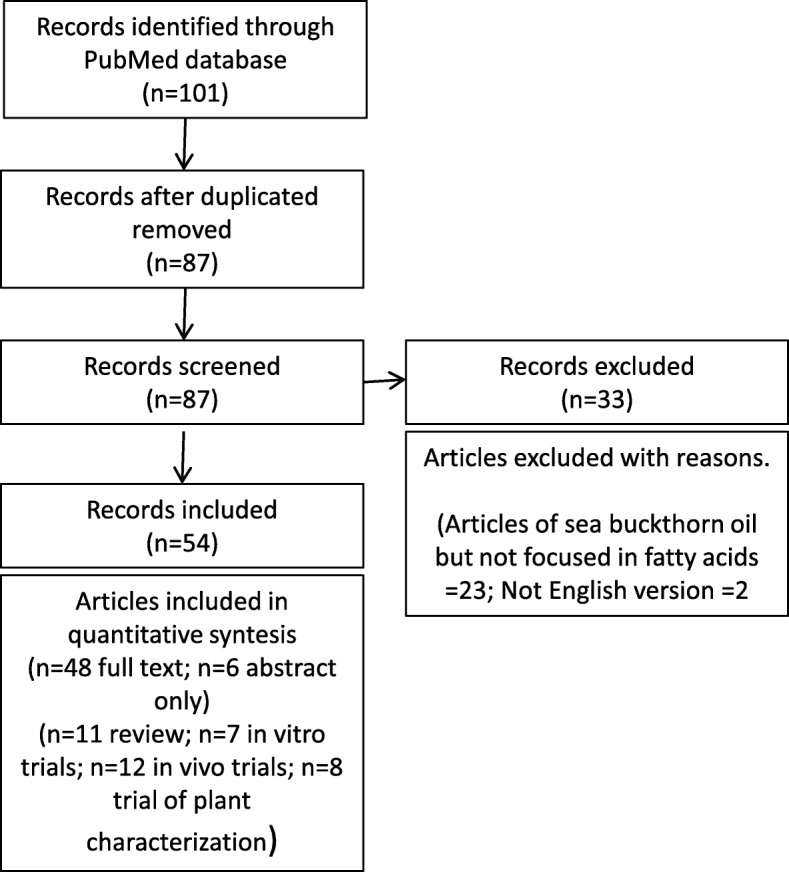
Literature search flow diagram

The systematic searches were conducted from April to June 2018. The search flow is summarized in Fig. 1. The first search includes 87 records after the duplications were removed. After the screening, 33 articles were excluded due to the fact that they did not focus on the fatty acids of sea buckthorn oil [10] and that they were written in Chinese [2]. The articles included (54) were reviews [11], in vitro trials [7] *in vivo* trials [12] and studies seeking to determine the qualitative and quantitative composition of sea buckthorn oil [8].

For the sake of clarity, the present review has been structured in three main parts, covering the three fatty acids found in sea buckthorn oil: firstly, monounsaturated fatty acids omega-7 and omega-9 (palmitoleic acid, vaccenic acid and oleic acid), then, polyunsaturated fatty acids omega-3 and omega-6 (linoleic acid and linolenic acid), and finally, the saturated fatty acids (stearic acid and palmitic acid). Each of these subheadings explains in more detail the characteristics of the relevant fatty acid: its chemical structure, its proportion (%) in the oil and its effects over human health in order of significance.

### Monounsaturated fatty acids

#### Palmitoleic acid (PA)

PA (16:1) is an omega-7 monounsaturated fatty acid very uncommon within the plant kingdom. Consequently, it is very difficult to introduce it in the human diet through vegetable food sources. Sea buckthorn is one of the few plants that present this fatty acid. Specifically, PA is only found in the soft part of the berry [4]. According to Yang & Kallio [1] the oil obtained from the pulp and peel of the sea buckthorn contains high levels of PA –up to 43%– [1]. On the contrary, the seed of the sea buckthorn presents extremely low levels of PA (< 0.5% of PA in triacilglicerol (TAG)) [13]. The composition of the fatty acid may be different according to its origin, subspecies or harvesting time. Some studies reveal that sea buckthorn originated in Central Asia and the Baltic region contained a higher proportion of PA in the TAG of the berry mescocarp than the ones originated in the Caucas. Another study compares the composition of omega-7 fatty acids in pulp/peel (palmitoleic and vaccenic acids) between the *subsp. sinensis* and *subsp. rhamnoides,* the latter presenting a higher proportion of it [1]. In addition, the proportion of vaccenic acid seed oil is very low compared to the rest of fatty acids (2–5%) [4].

##### Skin and mucous disorders

Several researchers conclude that PA takes part in different mechanisms that improve skin and mucous disorders. The main positive effects of PA are resumed in Table 2. Several studies emphasize the beneficial effects of PA for vaginal inflammatory atrophy treatment. Although an extensive range of treatments exist for this disorder –such as systemic and local estrogens, local testosterone, systemic and local corticoids, retinoid, anti-inflammatory ointment or surgery– these treatments are frequently inefficient. For this reason, alternative options such as the sea buckthorn application have been researched. For instance, a study published on Agro Food Industry Hi-Tech focuses on the oral administration of 3 capsules of omega-7 sea buckthorn to 5 patients twice a day during 12 weeks. The results of the study show a considerable improvement of chronic vaginal inflammatory atrophy in three cases. However, in the two least severe cases the improvement was less obvious. The mechanisms of omega-7 did not take place through an increase of circulating estrogen level, and it could therefore be an alternative to this treatment. Nonetheless, the number of patients recruited was small and more clinical trials are needed [11].

**Table 2:** Effects of Palmitoleic acid in vivo and in vitro clinical trials

Disorder	Study	Suggested mechanism	References
Vaginal inflammatory atrophy	Human in vivo study	The mechanism is unknown but it reflects a positive increasing trend of the vaginal health index without an increase of circulating estrogen level	Erkkola & Yang (2003) [11] Limo (2004)
Skin hyperpigmentation	In vitro study	Decrease hyperpigmentation though the Inhibition of tyrosinase, TRP-2 and MITF	Yoon (2010) [12]
Wounds infections	In vitro study	Take part in the growth of C.albicans decreasing its ability of adhesion in the skin and prevent the attachment of pathogenic yeast cells.	Wille & Kydonieus (2002)
Bacterial infection	In vitro study	PA calcium salt has a bactericidal effect against *S. aureus* and *P. acnes.*	Yamamoto (2015) [7]
Hypercholesterolemia	Mice in vivo study	A possible mechanisms may be related to the increase of LCAT	Budijanto (1992) [14]
Diabetes and liver dysfunctions	Mice in vivo study	It stimulate the uptake glucose and impaired the lipogenesis in liver by activation of AMPk and FGF-21, depending on PPAR-α	Souza (2016) Yang & Zhi-Hong (2010)

Another study focusing on the vaginal inflammatory atrophy suggested a daily oral intake of 3 g of sea buckthorn oil, where the main fatty acid was PA (24%) [12]. A total of 116 postmenopausal women were randomized into two groups: in the first one they were administered sea buckthorn, while in the second one they were administered placebo. The results of the study indicate a positive increasing trend of the vaginal health index for the sea buckthorn group [15]. Furthermore, the ability of PA is thought to inhibit melanogenesis. The melanogenesis is the synthesis of melanocyts, cells that produce the melanin pigment. The study of Yoon and colleagues [12] tried to determine the inhibition activity of the PA versus three melanogenic keys enzymes in murine B16 melanoma cells. These enzymes were tyrosinase, tyrosinase-related protein-1 (TRP-1), tyrosinase-related protein-2 (TRP-2) and microphtalmina-associated transcription factor (MITF). The results showed an inhibition of tyrosinase, TRP-2 and MITF, and consequently PA is proposed as a potential anti-melanogenic agent and effective in hyperpigmentation disorders [12].

Another effect of PA in skin and mucous is related to the human sebum. Human sebum consists mostly of triglycerides, wax esters and squalene, and its composition is characteristic of and unique to sebaceous cells [16]. Sebum does account for the self-disinfecting activity at the skin surface, and PA accounts for most of this activity. Wille & Kydonieus (2002) carried out an in vitro study based on collected skin surface lipids and investigated its antimicrobial activity. The results revealed that the PA at concentrations below 0.5 mg/ml had little effect on the growth of *C. albicans* and at concentrations above 1.0 mg/ml were effective in preventing the attachment of pathogenic yeast cells to isolated sheets of mammalian stratum corneum. Due to its potential activity against the adhesion of *C. albicans* to the skin, PA is included as a medical preventive treatment for wound infections and catheter coatings. However, this fatty acid is not effective in gram-negative bacteria. The authors highlight the possibility that PA may be a natural antimicrobial gram-positive bacteria of the skin [17]. Yamamoto and colleagues [7] investigate the bactericidal activity of PA calcium salt. By means of an in vitro study it was demonstrated the activity of this fatty acid against *Staphylococcus aureus* and *Propionibacterium acnes*. The selective bactericidal ability of PA calcium salt is a suitable property for an ingredient of cosmetic products because, in some cases, a higher bactericidal action affecting all types of bacteria is not always the best option for human skin [7].

##### Cholesterol levels

Moreover, PA seems to have an important role in lowering cholesterol levels. A study compares the effects of diet in regard to hypercholesterolemia in rats. The rats were distributed in six groups with different diets rich in either palmitate, stearate, palmitoleate, oleate, linoleate or α-linoleate. The palmitoleate was found to be as effective as linoleate in lowering the plasma cholesterol level. In addition, when it was compared to oleate and to other saturated acids, the palmitoleate was clearly more hypercholesterolemic. The mechanism of its activity remains unknown, but since there has been observed a high activity of lecitin cholesterol acil transferasa (LCAT) in rats fed with palmitoleate [14], the authors hypothesized that it may be associated to an increase of LCAT activity in plasma. In addition, a controlled trial with PA associated PA to a significant decrease in TAG and LDL-cholesterol, as well as to a significant increase in HDL-cholesterol in subjects with hyperlipidemia compared to the control group over 30 days [18].

##### Insulin resistance and liver dysfunction

Another effect of PA investigated in the study of Souza and colleagues (2016) is the possible relation between PA and hepatic insulin sensitivity. The design of the study consists in two groups of mice, one of them were fed a high-fat diet and the other a standard diet for 12 weeks. In the last 2 weeks, the high-fat diet mice were treated daily with oleic acid or palmitic acid. After 12 weeks, the mice were injected with insulin or vehicle and various parameters were analyzed. The study concluded that PA supplementation in mice fed with high-fat diet stimulated the uptake of glucose and impaired the lipogenesis in liver by activation of AMPk and FGF-21, dependent on PPAR-α. All these effects are essential to control insulin resistance and to reduce the ectopic deposition of lipids in the liver. The study also suggested that PA plays an important role as a non-pharmacological treatment for diabetes and liver dysfunctions. Moreover, it highlighted an increased lipolysis and a decreased lipogenesis in adipocytes. Additionally, the immunity system was seen to be affected by PA, which decreased NF-kB p65 phosphorylation and proinflammatory cytokine expression in macrophages [19]. Another study demonstrated that there is an increased insulin sensitivity in the liver and muscle, which improved hypertriglyceridemia and hyperglycemia in diabetic rats when they were fed 300 mg/kg of PA daily for 4 weeks [20]. More studies show a relation between the PA and the immunity system. This fatty acid promoted a suppressive effect in isolated human lymphocyte proliferation characterized by a decrease of Th1 and Th17 response, and co-stimulatory molecule (CD28) [21].

#### Oleic acid (OA)

OA (18:1) is a monounsaturated omega-9 fatty acid with 18 carbons and a double bound situated in the carbon number 9. Seed and soft parts are rich in oleic acid. The proportions of OA in both parts are 13–19% in seed oil and 12–33% in pulp oil [22]. An advantage of OA in regard to other monounsaturated fatty acids is its resistance to oxidation [23].

Although olive oil is the most well-known oil containing a high proportion of this fatty acid, there are other oils originating from plants such as sea buckthorn in which OA is also abundant. The Mediterranian diet is rich in OA due to the fact that one of the principal sources of fat is the olive oil, and there exist a strong association between a Mediterranean-style diet and protection for cardiovascular disease. Several studies show that OA decreases the expression of cell adhesion molecules in the endothelium [10], and this may be related to its antiatherogenic effects [24]. Carluccio and colleagues (1998) research the effect of OA on human umbilical vein endothelial cells in an in vitro study. The results reveal that OA works as an inhibitor of endothelial activation reducing the atherosclerotic risk. A possible mechanism to explain these antiatherogenic properties is the effect of OA in the expression on monocyte adhesion accounted for by an inhibition of mRNA and, upstream, by inhibition of NF-kB activation after endothelial cell stimulation. In addition, other mechanisms related on the lipid profile or other cardiovascular risk factor may exist [25].

### Polyunsaturated fatty acids

#### α-Linolenic acid (ala)

ALA (18:3) is an unsaturated omega-3 fatty acid composed by 18 carbons and three *cis* double bonds. It must be acquired through diet because it is an essential fatty acid and it is an isomer of GLA. It is found mostly in seed oil – 20-35% – and even though it is also present in the soft parts of the berries, the proportion is lower [4]. As in the other cases of aforementioned fatty acids, the proportion of ALA is different according to the subspecies. ALA shows a variation, with a higher average proportion in *subsp. rhamnoides* than *subsp. sinnensis.* [1]. ALA and GLA have an important role in the human organism (Table 3). They are a physiological component of cell membranes and mitochondria membranes and they play a role in the mechanism of cell transportation and the transmission of neuronal signals [31].

**Table 3:** Effects of linolenic acid in vitro and in vivo clinical trials

Disorder	Study	Possible mechanism of action	References
Cardiovascular risk	Human in vivo study	Ability to form EPA and DHA Reduction of plaque calcification Maintenance of endothelial function Reduction of blood pressure Exhibition of antithrombotic, anti arrhythmic and anti-inflammatory effects	Rajaram (2004) [26] Djoussé (2005) [27] Zhao (2004) [10] Takeuchi (2007)
Dry eye	Human in vivo study	Attenuation of the increase in tear film osmolarity and possible influence over the intensity of burning and redness symptoms	Larmo (2010) [28]
Fracture risk	Human in vivo study	Important role in bone turnover and maintenance of bone formation	Griel (2007) [29] Rajaram (2014) [26] García (2018) [30]

##### Cardiovascular disorders

The potent cardio protective activity of ALA has been researched in many studies [32]. EFSA authorized a health claim which affirms the contribution of ALA to the maintenance of normal blood cholesterol levels [33]. It is suggested that this effect is due to the ability of ALA to form precursors such as eicosapentaenoic acid (EPA) and docosahexaenoic acid (DHA) in the human body. However, ALA may have an impact upon health independently to its precursor role. Its cardio protective effects may be due to various mechanisms, such as the reduction of plaque calcification and the number of lipids, the maintenance of endothelial function, the reduction of blood pressure and the exhibition of antithrombotic, anti arrhythmic and anti-inflammatory effects [26].

Several clinical trials have tried to demonstrate all these effects. Djoussé and colleagues [27] carried out a cohort study where there was associated an inverse and dose-dependent relation between dietary ALA (range, 0.17 to 3.48 g/d) and prevalent calcified atherosclerotic plaque in the coronary arteries (CAC). The presence of CAC is directly associated with the total burden of atherosclerotic plaques and its vulnerability. Consequently, the reduction of the presence of CAC decreases the risk of atherosclerosis, and viceversa [27]. Furthermore, a cross-over study with moderate hypercholesterolemic subjects was conducted to prove the influence of the diet in the inflammation process in cardiovascular disease. The three diets designed in this study were: American diet (control diet), a diet high in polyunsaturated fatty acids and ALA (ALA diet) and a diet high in polyunsaturated fatty acids and LA (LA diet). The subjects were assigned to a sequence of these 3 test diets: they consumed a diet for 6 weeks and before starting the other diet they had a break period of 3 weeks. The results showed that ALA diet decreased C-reactive protein, a protein involved in the atherosclerotic process. Also, ALA diet decreased vascular cell adhesion molecule-1 (VCAM-1) and E-selectin compared to control diet. The 2 high-PUFA diets decreased serum total cholesterol, LDL cholesterol and TAG. In addition, ALA diet decreased HDL cholesterol and apolipoprotein AI more than American diet and increased EPA levels in serum. The research concluded that a diet rich in ALA plays a role in reducing multiple cardiovascular disease risk factors such as decreasing pro inflammatory cytokines, changes in VCAM-1, lowering cholesterol levels or increasing EPA levels in serum [10]. The anti arrhythmic mechanism of LA is postulated to be related to modulation of sodium and calcium channels of the cardio myocytes. Another cardio protective effect mentioned is the ability of ALA to reduce blood pressure. A study published in the *Journal of Olea Science* evaluated the antihypertensive activity of ALA in subjects with high-normal blood pressure and mild hypertension. The subjects were divided in two groups: a group which consumed 14 g of common blended oil and a group that intake ALA-enriched oil. The results revealed a significant decrease of systolic and diastolic blood pressure in the ALA-enriched group compared to the control group. The study concluded that ALA had an antihypertensive effect on blood pressure with no adverse effect during the study.

##### Dry eye

ALA is also related to several skin and mucous disorders. The actual treatments for the dry eye are not as efficient as they can be. For instance, artificial tears relieve symptoms without treating the causatives factors. Although anti-inflammatory drugs could produce side effects, they are still used to alleviate dry eye symptoms. In front of this situation, researchers are trying to investigate the potential effects of omega-3 fatty acids alone or with combination with omega-6 fatty acids in the treatment of dry eye. A randomized double-blind, parallel, placebo-controlled trial recruited a total of 100 volunteers to prove the beneficial effect of omega-3 in the treatment of dry eye. Two groups were formed and during 3 months (during the cold season) participants consumed 2 g of sea-buckthorn oil or placebo. According to the results, sea buckthorn oil attenuated the increase in tear film osmolarity and it may influence in the maximum intensity of burning and redness symptoms of dry eye [28].

##### Bone health

The potential effect of ALA regarding the protection of bone is being evaluated. A study assessed the relation of bone mineral density biomarker (N-telopeptides) and different diets. The results showed a significant decreased of N-telopeptids in the case of rich-ALA diet and rich-LA diet compared to the control diet. The study concluded that ALA and LA may play an important role in bone turnover and maintenance of bone formation [29]. Although these findings are encouraging, more robust data is needed. García and colleagues [30] also carried out a study among 1865 females to investigate the role of the diet in bone heath. Bone mineral density, Ward’s triangle, femoral trochanter, hip and lumbar were measured by X ray. The dietary intakes of total energy, calcium, vitamin D, ALA, EPA, DHA, LA and arachidonic acid were assessed by a food questionnaire. The authors found positive correlations between ALA, EPA and DHA with bone mineral density [30].

#### Linoleic acid (la)

LA (18:2) is a polyunsaturated omega-6 fatty acid with two double bounds in the 9 and 12 carbons. Together with α-linolenic acid (ALA), LA constitutes the essential fatty acids, which the human body cannot synthesize by itself. The European Food Safety Authority (EFSA) authorized a health claim stating that LA and ALA are needed for the normal growth and development of children [34].

LA is the major fatty acid in seed oil – up to 42% –, being also present in the berry and pulp oil in lower concentration levels (6 to 33%). There are small variations of concentration in seed oil according to the subspecies, being higher in the *subsp. rhamnoides* than in *subsp. sinensis.* [1] In addition, sea buckthorn oil is the only oil that naturally presents a 1:1 ratio of omega-3: omega-6 [35].

##### Skin condition

LA is the most abundant polyunsaturated fatty acid in human skin [36]. Consequently, many studies have focused on the beneficial effects of LA in mucous membrane and skin (Table 4). The lamellar granules of human skin produce lipids that maintain the protective barrier of the skin in order to avoid the epidermal loss of water. Zielsinska and colleagues (2014) affirm that the reproduction of lamellar granules slows down with aging, the skin becoming drier and weaker [31]. Omega-6 might reverse this process by accelerating the production of lipids in lamellar granules, strengthening the lipid barrier of the epidermis, protecting the skin against epidermal loss of water, and normalizing the metabolism of the skin [37]. In addition, people with acne skin present a decrease of LA in sebum, which leads to block pores and formation of comedos and eczema. In addition, it is thought that LA might improve sebaceous gland activity, unblocking pores and decreasing the number of comedos [31].

**Table 4:** Effects of linoleic acid in vitro and in vivo clinical trials

Disorder	Study	Possible mechanism of action	References
Skin disorders	Guinea pigs in vivo study	Transformation of LA to 13-HODE and consequently the modulation of coetaneous hyperproliferation	Ziboh (2000) [36]
Psoriasis	Human in vivo study	Reduce the rate of relapse and rebound	Min Liu (2015)
Atherosclerosis	Rabbits in vivo study	Decrease LDL-cholesterol levels	Larmo (2013) [15]

As mentioned above, a dietary deficiency in LA results in a characteristic scaly skin disorder and an excessive epidermal water loss. The physical structure of the epidermal water barrier is defined as a lipid bilayer, which fills the intracellular spaces of the upper-most layer of the epidermis (stratum corneum). This lipid bilayer contains sphingolipids; in turn, those sphingolipids rich in linoleate are called acylglucosylceramide, acylceramide and acylacid. Although the feeding of LA in fatty acid deficient animals is known to reverse the major coetaneous symptoms, the mechanisms by which this happened remain unknown. A possible mechanism is the transformation of LA into 13-hydroxioctadecadienoic acid (13-HODE). This reaction may be produced by incubated LA with soybean 15-lipoxygenase or 15-lipoxygenase prepared from skin epidermis in vitro [36]. The same study also demonstrated that 13-HODE formed was incorporated into epidermal phosphatidylinsotiol 4, 5-biphosphate, resulting in epidermal phospholipasa C-catalyzed release of 13-HODE into a novel 13-HODE-containing diacylglycerol. These results were obtained thanks to an in vivo trial in which Guinea pigs were randomized in three groups depending on diet: safflower (control), essential fatty acid deficiency diet, and essential fatty acid diet followed by safflower oil during 2 weeks. The third group showed replenished tissue concentrations of 13-HODE after the introduction of safflower oil, which inversely correlated with the selective down-regulation of protein kinase Cβ. It is possible that this 13-HODE-containing-diacylglycerol could function to decrease the activity of epidermal protein kinase C and epidermal hyperproliferation and differentiation. These results suggest that the epidermal concentration of 13-HODE, which is derived from dietary LA, may play a role in modulating coetaneous hyperporliferation [36].

Furthermore, LA seems to have an important role in the de novo biosynthesis of the major sebaceous fatty acids. An in vitro study of fatty acids from a biopsy punches from human facial skin rich in sebaceous glands was designed to try and elucidate the metabolisms of sebaceous lipids. Most fatty acids are esterified into triglycerides or polar lipids in sebaceous glands but LA is processed in a different way. It is hypothesized that LA is transformed into hydroxyperoxide by the lipoxygenase enzyme and this intermediate form is highly susceptible to β-oxidation. The results showed a correlation between the function and differentiation of sebaceous cells and the oxidation of LA. The study highlighted that the oxidation of LA is indispensable for the de novo biosynthesis of the major sebaceous fatty acids, such as palmitic and oleic acids [16].

Moreover, the positive effect of LA on psoriasis disease has also been considered. Min Liu and colleagues (2015) carried out a randomized study of 116 patients. The study was composed in two parts. In the first one the patients were divided in two groups. In one of them (the control group) patients were treated topically with 500 mg of a moderate corticosteroid cream. In the other, patients were treated with a LA-ceramides containing moisturizer in addition to the cream. After this first stage, the patients treated with LA-ceramide were randomized again. During this period, one part of the group maintained the use of moisturizer while the other group discontinued it. The study compared the rates of relapse and rebound between these new groups, being more favorable in the group that maintained the use of moisturizer. Based on the results obtained, the researchers concluded that the topical LA-Ceramide moisturizer could alleviate psoriasis, potentially becoming a valuable tool for its treatment and prevention [9].

##### Atherosclerosis

Another beneficial effect recently investigated is the relation between LA and cholesterol levels. Basu and colleagues [23] carried out a study where white rabbits with atherosclerosis were divided into two groups according to the diet that they were being administered. The first group was fed with 1 ml of seed oil of sea buckthorn, the second group was fed with a high-cholesterol diet, the third group was fed with high-cholesterol diet and after 30 days was administrated 1 ml of sea buckthorn oil, and the fourth was the control group. The results of the study indicated a significant decrease of LDL-cholesterol levels and atherogenic index in group 3 after the administration of the seed oil [23]. The study attributed these effects to LA but other authors considered that the lowering of serum cholesterol, LDL-cholesterol, triglycerides and other risk factors of cardiovascular disease have been associated not only to intakes of LA but also ALA, PA and sterols of sea-buckthorn oil [15].

#### γ-Linolenic acid (GLA)

GLA (18:3) is originated from a transformation of LA by a Δ-6-desaturasa. This fatty acid is an omega-6 and it is interesting due to the several effects it has on the organism. Many studies have focused on the benefits of omega-6 oils, particularly on GLA. Oils rich in GLA are reported to have positive benefits on skin conditions such as dermatitis and eczema, rheumatoid arthritis, pre-menstrual syndrome and the prevention of heart diseases [38](Table 5). Furthermore, it improves blood circulation, which is important for the nourishment and oxygenation of the skin, as well as to remove the excess of toxins. GLA is also a component of skin and is responsible for binds epidermis cells, being part of the intracellular cement of the skin. In addition, it is part of phospholipids, the major components of cell membranes. Other relevant properties of GLA are the capacity to protect skin against infections, counteract allergies, relieve inflammations, and slow down the ageing process [42].

**Table 5:** Effects of γ-linolenic acid in vitro and in vivo clinical trails

Disorder	Study	Possible mechanism of action	References
Acne	Human in vivo study	Forming products with anti-inflammatory properties through the cyclo-oxygenase and 15-lypogenase. Also, it can improve the hyperproliferative skin condition modulating the protein kinase C.	Jung (2014) [8]
Atopic dermatitis	Human in vivo study	It seems to play na important role as a marker of compliance but not as a therapeutic treatment	Jaw (2003) Yang (2000) [39]
Dry eye	Human in vivo study Rat in vivo study	In combination with omega-3 fatty acids, it decreases the secretion turbidity, the meibomian gland obstruction and edema.	Pinna (2007) [40] Viau (2009) [41]

##### Acne and atopic dermatitis

In relation to skin disorders, Jung and colleagues’ [8] study suggested that GLA could improve acne disease. Forty-five acne skin patients were divided in two different groups according to diet supplement. The supplements were omega-3 (group 1), GLA (group 2) and control group. The results obtained showed a significant reduction of the mean of inflammatory and non-inflammatory acne lesion in the groups 1 and 2 compared to the control group. Visual analogue scale was established at the beginning of the study, and it was also reduced in these two groups. Furthermore, interleukin 8, a type of interleukin presented in high levels in acne lesions [43], decreased significantly in the treatment groups. The study concluded by proposing two GLA possible mechanisms that may improve *acne vulgaris.* The first one consists in modulating the inflammatory process. GLA is converted in dihome-γ-linolenic acid (DGLA), a substrate of cyclo-oxygenase and 15-lypoxygenase. These two enzymes catalyze the production of prostaglandin E1 and 15-hydroxydihomo-γ-linolenic acid (15-OH-DGLA), which have anti-inflammatory properties. In the second one, 15-OH-DGLA can improve the hyper proliferative skin condition by the modulation of the protein kinase C [8].

The beneficial effects of PUFA on atopic dermatitis have been discussed for almost century. A double-blind, randomized-controlled trial investigated the effect of supplementation of borage oil (containing 100 mg of GLA) in infants with a maternal history of atopic disease. In this case, the study was not based on trying to elucidate the therapeutic effects in relation to atopic disease; instead, it focused on a possible protective role of GLA in the development of atopic dermatitis in infants with atopic mothers. The results showed an increase of GLA and phospholipids concentration in plasma in the treatment group, which were negatively associated with the severity of atopic dermatitis when infants were 1-year-old. However, there were not significant differences in total serum IGE concentration between the two groups. The study concluded that the supplementation of GLA as a treatment seemed to be favorable but not significant. Instead, when GLA is used as a marker of compliance, the severity of AD is strongly negatively associated with increase of GLA in infants. Finally, the authors suggested that an early supplementation with GLA in children tend to alleviate the severity of atopic dermatitis in later infancy [44]. In accordance with these outcomes, various studies have tried to relate the effects of PUFA to atopic dermatitis as a therapeutic treatment, but their results were not conclusive [39].

##### Meibomian gland dysfunction

In regard to dry eye disorders, a study researched the effect of ALA and GLA on meibomian gland dysfunction. The meibomian glands are situated inside the eyelids, and when a blockage of this gland exists, the oily part of the tears cannot be released, producing an acceleration of the drying of the eyes and generating irritation [45]. Pinna and colleagues [40] designed a study where subjects were divided into 3 groups. A group consumed ALA (28,5 mg) and GLA (15 mg) tablets; B group was subjected to eyelid hygiene and C group received both treatments. All treatments were once daily for 180 days. Although the three treatments presented a significant improvement in symptoms, the C group was the most efficient. The administration of GLA and LA (A group) decreased the secretion turbidity and the meibomian gland obstruction. When it was combined with eyelid hygiene (C group) it showed, in addition, a reduction in eyelid edema, foam collection in the tear meniscus and corneal staining. The study suggested a dual effect of omega-6 fatty acids in the treatment of meibomian gland dysfunction. On the one hand, the anti-inflammatory activity may contribute to reducing eyelid edema. On the other, it may produce a less dense and turbid meibomian secretion by modifying the lipid composition. Thus, it may increase the efficacy of eyelid hygiene [40]. Another study carried out with a dry eye rat model revealed that dietary polyunsaturated fatty acids prevented the clinical signs of chronic corneal dryness and prevented the loss of musincs in the conjunctival epithelium. Only the rats treated with a combination of omega-6 and omega-3 decreased the expression of MHC II (inflammatory marker) on the conjunctival epithelium. The authors concluded that a combination of omega-3 and GLA fatty acids may be more successful in the treatment of dry eyes than the application of only one of them [41].

### Saturated fatty acids

The proportion of palmitic acid (16:0) was higher in subsp. *sinensis* than subsp. *rhamnoides.* On the contrary, stearic acid (18:0) has a major proportion in *subesp rhamnoides* [1]. The proportion of palmitic acid is quite high in seed oil −15 to 20%– while in the case of stearic acid is very poor 2–4% [4].

Saturated fatty acids (stearic and palmitic) are not known to reveal a beneficial effect in human health if they are taken orally. However, they may be interesting if they are administrated topically due to its molecular structure. Saturated fatty acids are characterized for not containing any double bond in its chain. This distinctive aspect affects in its form, tending to be more solid than unsaturated fatty acids, and consequently being more occlusive. Its occlusive properties may be beneficial in skin disorders that develop dehydrated skin. Thanks to this property, saturated fatty acids form a protective barrier in the skin valuable in clinical application for two different reasons. On one hand, the barrier formed prevents the skin loss of water and, on the other hand, it facilitates the absorption of other bioactive compounds of the oil, such as the rest of fatty acids.

In addition, they have a remarkable effect in organoleptic aspects. They are more resistant to oxidation than unsaturated fatty acids, and, for this reason, they confer to the oil a high stability. Moreover, the presence of these fatty acids provides excellent texture properties to the oil such as smoothing and softening [46], being more attractive to the consumers.

## Conclusion

Sea buckthorn oil is very rich in fatty acids and could play an important role in several activities related to human health. PA has an evident clinical application on skin and mucous disorders such as vaginal inflammatory atrophy, skin hyper pigmentation or wounds, infections. In addition, other different positive effects in hypercholesterolemia, diabetes and liver dysfunction are demonstrated. The OA has a well founded indication in the protection of cardiovascular diseases. Although ALA is known to reduce cardiovascular risk, it is also useful in dry eye and health bone. The omega-6 fatty acids (GLA and LA) may have clinical applications in skin disorders. LA is beneficial in psoriasis and GLA in acne skin, atopic dermatitis and dry eye. In addition, LA seems to improve atherosclerosis condition. Large amount of experimental data evidencing those fatty acids could influence in a huge range of activities in human health being a possible candidate for several clinical application. It is possible to conclude that sea buckthorn oil is a promising product due to its diversity of fatty acids and its unique composition of omega-7 fatty acids group and these fatty acids have a strong relation with human health. However, the main results of the present review are obtained from studies of fatty acids isolated due to the lack of researches of sea buckthorn oil fatty acids. Although these studies permit to extrapolate the effects of the isolated fatty acids in sea buckthorn oil, they limit the possible synergic effect between the fatty acids or with other compounds present in the oil.

## Abbreviations

13-HODE: 13-hydroxioctadienoic
15-OH-DGLA: 15-hydoxydihomo-γ-linolenic acid
ALA: α-linolenic acid
CAC: Calcified coronary arteries
DGLA: Dihome- γ-linolenic acid
DHA: Docosahexaenoic acid
EFSA: European Food Safety Authority
EPA: Eicosapentaenoic acid
FGF-21: Fibroblast growth factor 21
GLA: Gamma-linolenic acid
HDL: High density lipoprotein
LCAT: Lecithin cholesterol acil transferasa
LDL: Low density lipoprotein
MITF: Microphtalmina-associated transcription factor
OA: Oleic acid
PA: Palmitoleic acid
PPAR-α: Peroxisome proliferator-activated receptor alpha
PUFA: Polyunsaturated fatty acids
Subsp: Subspecies
TAG: Triacilglicerol
TRP-1: Tyrosinase-related protein-1
TRP-2: Tyrosinase-related protein-2
VCAM-1: Vascular cell adhesion molecule-1
